# The influence of incubation temperature on offspring traits varies across northern and southern populations of the American alligator (*Alligator mississippiensis*)

**DOI:** 10.1002/ece3.10915

**Published:** 2024-02-15

**Authors:** Christopher R. Smaga, Samantha L. Bock, Josiah M. Johnson, Thomas Rainwater, Randeep Singh, Vincent Deem, Andrew Letter, Arnold Brunell, Benjamin B. Parrott

**Affiliations:** ^1^ Eugene P. Odum School of Ecology University of Georgia Athens Georgia USA; ^2^ The University of Georgia's Savannah River Ecology Laboratory Aiken South Carolina USA; ^3^ Belle W. Baruch Institute of Coastal Ecology and Forest Science Clemson University Georgetown South Carolina USA; ^4^ Tom Yawkey Wildlife Center Georgetown South Carolina USA; ^5^ Fish and Wildlife Research Institute, Florida Fish and Wildlife Conservation Commission Gainesville Florida USA

**Keywords:** developmental plasticity, life history, maternal provisioning, temperature‐dependent sex determination

## Abstract

Maternal provisioning and the developmental environment are fundamental determinants of offspring traits, particularly in oviparous species. However, the extent to which embryonic responses to these factors differ across populations to drive phenotypic variation is not well understood. Here, we examine the contributions of maternal provisioning and incubation temperature to hatchling morphological and metabolic traits across four populations of the American alligator (*Alligator mississippiensis*), encompassing a large portion of the species' latitudinal range. Our results show that whereas the influence of egg mass is generally consistent across populations, responses to incubation temperature show population‐level variation in several traits, including mass, head length, head width, and residual yolk mass. Additionally, the influence of incubation temperature on developmental rate is greater at northern populations, while the allocation of maternal resources toward fat body mass is greater at southern populations. Overall, our results suggest that responses to incubation temperature, relative to maternal provisioning, are a larger source of interpopulation phenotypic variation and may contribute to the local adaptation of populations.

## Graphical abstract

The influence of incubation temperature, not maternal provisioning, varies across populations of the American alligator. Responses to incubation temperature are a larger source of interpopulation trait variation relative to maternal provisioning and may be more likely to be modified by selection.

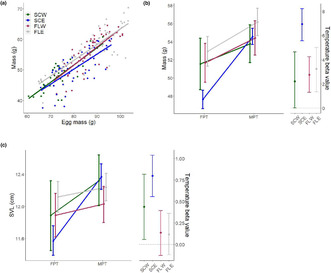

## 1 INTRODUCTION

Developmental plasticity, the expression of alternative phenotypes under different environmental conditions, is a fundamental driver of phenotypic variation across organismal and population‐level scales. Organismal responses to the developmental environment can be adaptive, neutral, or mal‐adaptive (Forsman, 2015; Ghalambor et al., 2007), having important implications for both ecology and evolution (Miner et al., 2005; West‐Eberhard, 1989, 2003). For example, when conditions experienced during development provide reliable cues of later life environments, developmental plasticity can be adaptive by maximizing phenotype‐environment matching (Nettle & Bateson, 2015; Pfennig, 1990). Alternatively, environments that disrupt normal developmental processes can lead to plastic responses with negative effects on fitness (Barker, 2001; Guillette et al., 1995), while physical constraints on development can give rise to plasticity that is neutral with respect to fitness (Ghalambor et al., 2007; Gotthard & Nylin, 1995). Regardless of their adaptive value, many embryonic responses to the developmental environment have a heritable, genetic basis and can vary, suggesting they can evolve under novel selective pressures (Pigliucci, 2005).

In oviparous vertebrates, maternal provisioning of nutrients and signaling molecules is critical for proper development and can be a major determinant of offspring traits (Groothuis et al., 2005; Radder et al., 2007; Van Dyke & Griffith, 2018). Complex biological and ecological factors, including maternal diet (Royle et al., 2003; Warner & Lovern, 2014), stress (McCormick, 1998; Saino et al., 2005), and age (Beamonte‐Barrientos et al., 2010; Urvik et al., 2018), can influence the quantity and quality of resources provisioned to embryos (Moore et al., 2019; Mousseau & Fox, 1998). However, other components of the developmental environment can influence how maternal resources are utilized by developing embryos (Brown et al., 2011; Du & Shine, 2008, 2022; Mueller et al., 2015; Shine & Brown, 2002). For example, egg mass is a primary determinant of hatchling mass (Deeming & Birchard, 2007), but incubation temperature has been shown to influence diverse hatchling phenotypes across many species (While et al., 2018). This includes modifying the efficiency by which maternal resources are converted into somatic tissue (Bock et al., 2021; Marshall et al., 2020; Pettersen et al., 2019) and how those resources are allocated to specific phenotypes (Flatt, 2001; Telemeco et al., 2010). However, despite the importance of maternal provisioning and incubation temperature in modifying hatchling phenotypes, the extent to which responses to these factors vary across populations is not well resolved (but see Bodensteiner et al., 2019; Orizaola & Laurila, 2009; Orizaola & Laurila, 2016; Richter‐Boix et al., 2015).

When viewed through the lens of Developmental Cost Theory (DCT, Marshall et al., 2020), the influence of incubation temperature on maternal resource use represents a fundamental developmental constraint (Gotthard & Nylin, 1995). According to DCT, the energy required for development can be quantified as the product of development rate and metabolic rate (Pettersen et al., 2019). Whereas temperature affects both developmental and metabolic rates, differences in their temperature‐dependence result in an optimal temperature at which developmental cost is minimized. As a result, environmental temperatures typically encountered by embryos in nature are tightly correlated to species‐specific thermal optima that minimize developmental cost (Marshall et al., 2020; Pettersen et al., 2019). Deviations from these optima are predicted to decrease developmental efficiency and result in reduced size, growth, and energy reserves of individuals. Importantly, responses of metabolic rate and developmental rate to temperature can be decoupled (Pettersen, 2020; Williams et al., 2016). Therefore, thermal dependencies of metabolic and/or development rate can evolve independently, allowing selection to modify the temperature at which developmental cost is minimized under novel thermal environments (Pettersen et al., 2023).

In species with broad geographic ranges, divergent climatic conditions have the potential to exert novel selective pressures on traits influenced by the developmental environment (Conover & Schultz, 1995; Kawecki & Ebert, 2004; Merilä et al., 2000; Orizaola & Laurila, 2009). Populations inhabiting high altitudes and latitudes are often exposed to colder temperatures (Angilletta, 2009), which impose novel thermal constraints on development. To compensate, populations can adapt by altering the thermal sensitivity of developmental processes. For instance, in oviparous reptiles, cooler incubation temperatures can result in longer incubation duration. Embryos from high‐altitude and latitude populations compensate by displaying faster development rates when compared to those from lower altitudes or latitudes under identical incubation temperatures (Du, Warner, et al., 2010; Pettersen, 2020), regardless of egg size (Storm & Angilletta Jr, 2007). These opposing effects of genetic and environmental influences on developmental rate, known as counter‐gradient variation (Conover & Schultz, 1995), are thought to reduce the cost of development and allow more time for offspring to acquire resources prior to colder, harsher winters (Olsson & Shine, 1997; Pettersen, 2020). Similarly, high‐altitude populations of wall lizards (*Podacris uralis*) have been shown to allocate more maternal resources toward somatic tissue relative to low‐altitude populations when raised at a common temperature (Pettersen et al., 2023). However, our understanding of the extent to which populations vary in how maternal provisioning and incubation temperature shape fitness‐related traits in taxonomically diverse species is limited (While et al., 2018).

In the present study, we test whether populations vary in embryonic responses to maternal provisioning and incubation temperature in the American alligator (*Alligator mississippiensis*). The alligator's latitudinal range extends from southern Florida to northeastern North Carolina (Elsey et al., 2019), providing potential for local adaptation of phenotypic responses to the developmental environment. Few studies have examined variation in nest temperatures across the alligator's range, but comparisons between a northern and southern population did not find significant differences in mean nest temperature (Bock et al., 2020). However, this was based on only 3 years of overlap between populations, and within each year, the mean nest temperature of southern populations was greater than that of northern populations (Bock et al., 2020). Cooler temperatures at northern latitudes would presumably decrease developmental rate, increase the cost of development, and delay hatching dates, reducing time for resource acquisition prior to winter (Olsson & Shine, 1997). Despite these potential differences, there is little information on how responses to the developmental environment vary across the alligator's range.

Like many turtles and some lizards, alligators display temperature‐dependent sex determination (TSD), in which thermal signals experienced during a discrete developmental window determine sex, along with additional phenotypic traits (Allsteadt & Lang, 1995; Bock et al., 2021; Kohno et al., 2014; McCoy et al., 2016). Specifically, incubations at warmer, male‐promoting temperatures (MPT) reduce developmental costs, producing larger hatchlings with greater residual yolk reserves when compared to incubations at cooler female‐promoting temperatures (FPT, Allsteadt & Lang, 1995; Bock et al., 2023, Bock et al., 2021). Recent reports demonstrate that temperature‐sensitive traits, including body mass index (BMI) and snout‐vent length (SVL), are associated with higher juvenile survival at MPT in the alligator (Bock et al., 2023; Johnson et al., 2023). However, these appear to be context dependent as the relationship between phenotypic traits and survival varies across years (Bock et al., 2023). Nonetheless, given that warmer, MPT appears to be the optimum developmental temperature in this species, we hypothesize that northern populations, presumably exposed to cooler temperatures, will show compensatory responses to incubation temperature. Using a common garden incubation and grow out design, we resolve the relative influences of incubation temperature and maternal provisioning on aspects of developmental cost (hatchling mass and incubation duration), along with other morphological (SVL, tail girth [TG], head length [HL], head width [HW], and BMI) and metabolic (10‐day growth, residual yolk mass and fat body mass) traits across populations. We predict that northern populations will display greater mass and developmental and growth rates relative to southern populations at cooler incubation temperatures. Additionally, we predict that northern populations will have increased residual energy reserves (residual yolk mass and fat body mass), decreasing the need to acquire resources after development prior to winter.

## 2 METHODS

### 2.1 Experimental design and data collection

**FIGURE 1:**
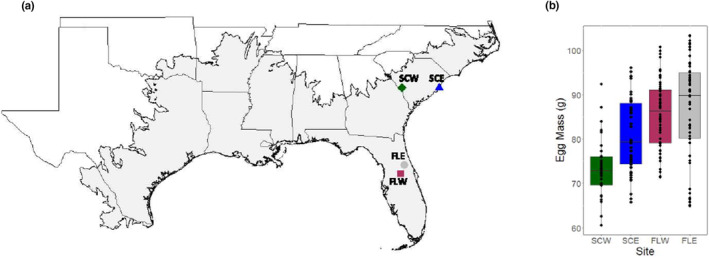
Geography and egg size of sampled populations. (a) Map showing the geographic range of the American alligator and sampled populations. (b) Egg mass variation across populations.

In June and July of 2021, 7–8 clutches (eggs from one nest originating from the same female) of alligator eggs were collected from each of four, geographically distinct populations (total *n* = 1378), including Par Pond on the United States Department of Energy's Savannah River Site in Aiken, South Carolina (South Carolina West, SCW), Tom Yawkey Wildlife Center in Georgetown, South Carolina (South Carolina East, SCE), Lake Woodruff National Wildlife Refuge in De Leon Springs, Florida (Florida East, FLE), and Lake Apopka in Apopka, Florida (Florida West, FLW; Figure 1a). After locating nests by helicopter or airboat, all eggs were removed from a nest cavity within 2 weeks of oviposition. Eggs were placed in plastic bus pans with nesting material from natural nests and driven back to the University of Georgia's Savannah River Ecology Laboratory (SREL) in Aiken, SC (within 4–24 h after egg collection), where they were individually weighed and 1–2 eggs from each clutch were staged according to Ferguson (1985) to determine stage at collection. The remaining eggs were transferred into new bus pans with dampened sphagnum moss and kept in commercial incubators (model I36NLC, Percival Scientific, Perry, IA, USA) at 32°C, an intermediate temperature that produces mixed sex ratios (Lang & Andrews, 1994). During this period, eggs were misted twice daily, and bins were rotated once daily within each incubator to limit the effect of intra‐incubator temperature variation. Incubator temperatures were also monitored with HOBO TidbiT® v2 Temp Loggers (Onset, Bourne, MA, USA).

Embryonic stage 15 (occurring approximately 15 days post‐oviposition), just prior to the opening of the thermosensitive period of sex determination (McCoy et al., 2015), was predicted based on the stage of eggs at collection and eggs from each population were randomly assigned in a split‐clutch design to one of two temperature treatments: a constant MPT (33.5°C) or a constant FPT (29.5°C). Since full clutches were collected for multiple studies, a random subset of 3–10 eggs/clutch/temperature/site were chosen at this time to raise until hatch for this experiment. Throughout the entire incubation period, eggs were continually monitored as above. While it is increasingly noted that constant temperatures may not be reflective of natural nest conditions (Bowden et al., 2014; Hall & Warner, 2020), the temperatures utilized here have been previously examined in the alligator with known effects on hatchling phenotypes, providing a basis with which to compare our results.

Once embryos pierced the eggshell (“pipped”), the date was recorded, and eggs were placed in glass Mason jars (one egg/jar) with damp, sphagnum moss. Embryos were given 48 h to hatch from the egg before being assisted if they did not hatch on their own. Once fully hatched, individuals were weighed using a digital balance (±0.01 g) and SVL and TG were measured using a flexible ruler (±0.1 mm), and HL and HW were measured using calipers (±1 mm). Hatchlings were then individually marked using unique, numbered toe tags and transported to large, indoor, fiberglass holding tanks where they were held at the SREL aquatic animal facility for 10 days. The aquatic animal holding facility is a semi‐climate controlled building with translucent fiberglass ceilings, mimicking natural light cycles and maintaining temperatures between 21 and 29°C (Johnson et al., 2023; Tuberville et al., 2016). During this period, hatching alligators relied on maternal yolk reserves and were not fed (Allsteadt & Lang, 1995). Water was changed daily (using tap water), and hatchlings were monitored visually twice daily for overall health and survival. At 10‐days post‐hatch (10‐DPH), hatchlings were remeasured, euthanized via cervical severance and pithing, and dissected to obtain residual yolk mass and fat body mass. Phenotypes analyzed included morphological traits of mass, SVL, TG, HL, HW, and body condition (BMI: mass/2*SVL) at hatch, and metabolic traits including incubation duration (measured in days from stage 15 to pip), change in morphological traits between 10‐DPH and hatch (∆ mass ∆ BMI, ∆ SVL, ∆ TG), residual yolk mass, and fat body mass. All experiments were approved by the University of Georgia Animal Care and Use Committee (A2021 05‐007‐Y3‐A0), and collections were carried out under permits from the South Carolina Department of Natural Resources (SC‐08‐2021) and Florida Fish and Wildlife Conservation Commission (SPGS‐18‐33).

### 2.2 Statistical analysis

All statistical analyses were conducted in RStudio (R Core Team, 2021, version 4.1.2), and all models were built using the *lme4* package (Bates et al., 2015). Model assumptions of residual normality and homoscedasticity were checked visually via residual vs fitted and Q–Q plots, with log transformations made for residual yolk and fat body mass to best meet assumptions. To compare initial egg mass across populations, we used a linear mixed‐effects model (LMM) including a fixed‐effect of site and random intercepts to control for clutch effects. To determine whether hatch probability or survival to 10‐DPH differed across temperatures or sites, we used a generalized linear mixed model with a binomial distribution including temperature, site, and their interaction as fixed effects, including random intercepts of clutches nested within sites. Post‐hoc pairwise comparisons were conducted using the *emmeans* package (Lenth et al., 2023) with Kenward–Roger degrees of freedom and correcting for multiple testing using Tukey's method.

To test for differences in the relative contributions of egg mass and incubation temperature to phenotypic traits across populations, we constructed separate LMMs for every phenotype at each site. In every model, we included fixed effects of egg mass and incubation temperature, while controlling for clutch effects using random intercepts. We then compared model estimates across populations by extracting beta values (i.e., effect size estimates) and 95% confidence intervals (CIs) using the confint function in R. Model beta estimates in which CIs did not overlap zero or another population were considered statistically significant.

To further examine how embryos respond to temperature and maternal provisioning across populations, we used the *ggeffects* package (Lüdecke, 2018) to predict temperature‐specific mean values of each phenotype at a common egg mass, corresponding to the average egg mass across the dataset (*x̄* = 82.75 g, SD = 9.99), from each population‐specific model. By comparing egg mass‐corrected mean phenotypes across temperatures and populations, we were able to determine whether populations differed in mean trait values irrespective of egg mass at either or both temperatures and whether variation in the influence of incubation temperature was driven by phenotypic differences at 29.5°C, 33.5°C, or both. Mean values in which 95% CIs did not overlap were considered statistically significant.

Given that populations can also vary in how maternal resources are allocated toward particular phenotypes, we compared ratios of SVL, TG, HL, HW, residual yolk mass, and fat body mass to hatchling mass across populations within and across temperatures using LMMs. For this analysis, we included temperature, site, and their interaction as predictors, along with egg mass as a covariate, controlling for clutches nested within sites using random intercepts. We then compared predicted mean values from the model within and among temperatures across populations using the *emmeans* package. Values in which CIs did not overlap were considered statistically significant. We used ratios of traits to hatchling mass instead of egg mass for this analysis because there were significant differences in temperature‐specific mass across populations (see below), and as a result, differences in the ratio of traits to egg mass would be confounded by population‐specific effects of temperature on mass and may not represent differences in the allocation of maternal resources toward specific phenotypes. All figures were created using the R package *ggplot2* (Wickham, 2016).

## 3 RESULTS

### 3.1 Egg mass and survival

Egg masses at the two southern populations (FLW: *x̄* = 85.1, SE: 3.08; FLE: *x̄* = 86.9, SE: 3.29) were greater relative to the two northern populations (SCW: *x̄* = 74.9, SE: 3.31 and SCE: *x̄* = 80.9, SE: 3.08), but only a nearly significant difference was observed between SCW and FLE (*β* = −11.98, *t* = −2.57, *p* = .073; Figure 1b). Whereas hatch rates were lower at 29.5°C (59.2%) compared to 33.5°C (82.9%; *β* = 1.12, *z* = 2.08, *p* = .038), differences were not observed between sites at either temperature (all pairwise *p* > .23). There were also no differences in survival between sites (all pairwise *p* = 1) or temperatures (*p* = 1) during the 10‐day growth period, with 79 (94%) and 114 (94%) animals surviving at 29.5°C and 33.5°C, respectively. Final sample sizes of surviving individuals by temperature, clutch, and site are shown in Table 1.

**TABLE 1:** Final sample sizes of surviving individuals by temperature and clutch.

Clutch	FLW		SCW		FLE		SCE	
	FPT	MPT	FPT	MPT	FPT	MPT	FPT	MPT
1	5	4	2	3	6	3	4	5
2	3	4	2	3	5	5	2	3
3	1	2	1	3	2	5	3	5
4	4	5	2	3	4	4	2	4
5	4	5	3	3	4	5	2	4
6	4	8	2	3	3	5	4	2
7	0	3	0	2	0	0	1	2
8	0	5	–	–	2	4	2	2
Total	21	36	12	20	26	31	20	27

### 3.2 Morphological traits

**FIGURE 2:**
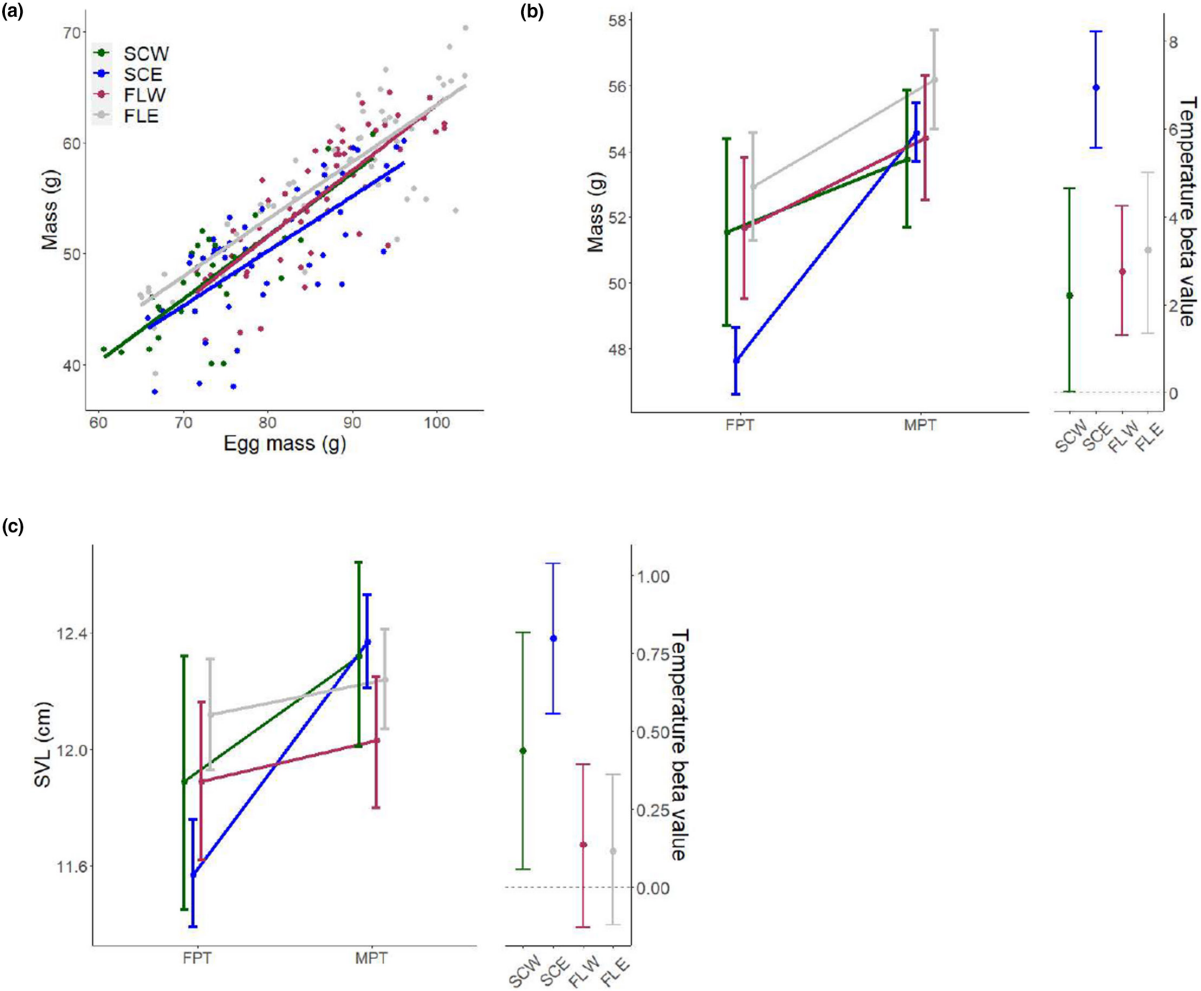
Population variation in the influence of egg mass and temperature on morphological traits, showing (a) the relationship between egg mass and hatchling mass, (b) hatchling mass, and (c) snout‐vent‐length (SVL). In (b) and (c), plotted values are model means under a common egg mass (82.75 g). Beta values are effect size estimates, and error bars represent 95% confidence intervals.

Both egg mass and temperature exerted positive effects on hatchling mass across all populations (Table 2). However, whereas the influence of egg mass did not differ across sites (Figure 2a), temperature more strongly affected hatchling mass at SCE compared to the other three populations (SCW: *β* = 2.223, CI = [0.035, 4.637]; SCE: *β* = 6.948, CI = [5.758, 8.224]; FLW: *β* = 2.755, CI = [1.314, 4.255]; FLE: *β* = 3.242, CI = [1.355, 5.004]; Figure 2b). In addition, there was a trend for a greater influence of incubation temperature on SVL at the northern populations relative to the southern populations, with the influence of temperature on SVL not significant in the latter (SCW: *β* = 0.438, CI = [0.058, 0.817]; SCE: *β* = 0.799, CI = [0.557, 1.041]; FLW: *β* = 0.136, CI = [−0.135, 0.395]; FLE: *β* = 0.117, CI = [−0.127, 0.361]; Figure 2c). Across other morphological traits, the influence of temperature was variable in both direction and magnitude, with significant differences between SCE and FLE for TG (SCE: *β* = 0.186, CI = [0.060, 0.314]; FLE: *β* = −0.091, CI = [−0.183, −0.004]), HL (SCE: *β* = 1.382, CI = [0.938, 1.825]; FLE: *β* = 0.001, CI = [−0.527, −0.462]), and HW (SCE: *β* = 0.176, CI = [−0.192, 0.544]; FLE: *β* = −0.670, CI = [−0.968, −0.373]). Meanwhile, the influence of egg mass was not different across populations for any trait (Table 2).

**TABLE 2:** Model results for the influence of incubation temperature and egg mass on phenotypes at each population. Superscripts for each beta value (effect size estimate) denote significant differences (non‐overlapping confidence intervals) between sites within each phenotype. Phenotypes in which at least one population comparison was significant are bolded.

Phenotype	Site	Temp *β*	Temp CI	Egg mass *β*	Egg mass CI	Clutch variance (SD)	Residual variance (SD)	*N*
**Mass**	SCW	2.223^a^	0.035, 4.637	0.523^a^	0.349, 0.698	1.192 (1.092)	8.404 (2.899)	31
**Mass**	SCE	6.948^b^	5.758, 8.224	0.551^a^	0.464, 0.633	0.265 (0.515)	4.295 (2.073)	48
**Mass**	FLW	2.755^a^	1.314, 4.255	0.477^a^	0.298, 0.664	5.698 (2.387)	6.349 (2.52)	57
**Mass**	FLE	3.242^a^	1.355, 5.004	0.515^a^	0.409, 0.619	1.237 (1.112)	11.111 (3.333)	54
**SVL**	SCW	0.438^ab^	0.058, 0.817	0.019^a^	−0.008, 0.046	0 (0)	0.251 (0.501)	29
**SVL**	SCE	0.799^b^	0.557, 1.041	0.029^a^	0.014, 0.043	0 (0)	0.181 (0.425)	49
**SVL**	FLW	0.136^a^	−0.135, 0.395	0.031^a^	0.009, 0.054	0.047 (0.216)	0.227 (0.476)	57
**SVL**	FLE	0.117^a^	−0.127, 0.361	0.025^a^	0.014, 0.036	0 (0)	0.212 (0.46)	54
**TG**	SCW	−0.041^ab^	−0.232, 0.15	0.018^a^	0.004, 0.032	0 (0)	0.068 (0.262)	32
**TG**	SCE	0.186^b^	0.06, 0.314	0.015^a^	0.008, 0.023	0.001 (0.031)	0.048 (0.219)	49
**TG**	FLW	−0.024^ab^	−0.143, 0.091	0.016^a^	0.002, 0.030	0.034 (0.185)	0.043 (0.206)	58
**TG**	FLE	−0.091^a^	−0.183, −0.004	0.018^a^	0.01, 0.025	0.011 (0.105)	0.026 (0.163)	54
BMI	SCW	0.044^a^	−0.023, 0.123	0.018^a^	0.013, 0.023	0.001 (0.029)	0.008 (0.09)	28
BMI	SCE	0.162^a^	0.115, 0.208	0.018^a^	0.014, 0.021	0.001 (0.035)	0.006 (0.076)	47
BMI	FLW	0.086^a^	0.027, 0.148	0.010^a^	0.002, 0.019	0.026 (0.16)	0.01 (0.101)	56
BMI	FLE	0.113^a^	0.042, 0.179	0.017^a^	0.013, 0.021	0.002 (0.045)	0.016 (0.126)	54
**HL**	SCW	0.720^ab^	−0.047, 1.488	0.038^a^	−0.018, 0.094	0 (0)	1.102 (1.05)	32
**HL**	SCE	1.382^b^	0.938, 1.825	0.051^a^	0.024, 0.078	0 (0)	0.614 (0.784)	50
**HL**	FLW	0.563^ab^	0.009, 1.067	0.070^a^	0.034, 0.107	0.057 (0.239)	0.953 (0.976)	58
**HL**	FLE	0.001^a^	−0.527, 0.462	0.049^a^	0.021, 0.075	0.072 (0.268)	0.792 (0.89)	54
**HW**	SCW	−0.460^ab^	−1.088, 0.167	0.037^a^	−0.008, 0.083	0 (0)	0.737 (0.858)	32
**HW**	SCE	0.176^b^	−0.192, 0.545	0.030^a^	0.007, 0.052	0 (0)	0.423 (0.65)	50
**HW**	FLW	−0.217^ab^	−0.582, 0.124	0.037^a^	0.004, 0.07	0.135 (0.367)	0.398 (0.631)	58
**HW**	FLE	−0.670^a^	−0.968, −0.373	0.042^a^	0.029, 0.055	0 (0)	0.313 (0.56)	54
**∆ Mass**	SCW	−1.063^a^	−1.861, −0.265	0.009^a^	−0.048, 0.065	0 (0)	1.127 (1.061)	31
**∆ Mass**	SCE	−0.643^a^	−1.255, −0.107	−0.010^ab^	−0.059, 0.041	0.278 (0.527)	0.851 (0.923)	48
**∆ Mass**	FLW	−0.112^a^	−0.644, 0.42	−0.086^b^	−0.12, −0.053	0 (0)	0.987 (0.993)	56
**∆ Mass**	FLE	−0.783^a^	−1.421, −0.121	−0.062^ab^	−0.101, −0.022	0.222 (0.471)	1.37 (1.171)	53
∆ SVL	SCW	−0.225^a^	−0.503, 0.025	0.006^a^	−0.016, 0.022	0.016 (0.127)	0.11 (0.331)	29
∆ SVL	SCE	−0.280^a^	−0.481, −0.093	−0.003^a^	−0.019, 0.013	0.023 (0.153)	0.107 (0.327)	49
∆ SVL	FLW	0.065^a^	−0.178, 0.275	0.003^a^	−0.016, 0.021	0.021 (0.146)	0.157 (0.396)	56
∆ SVL	FLE	−0.027^a^	−0.217, 0.165	0.005^a^	−0.005, 0.014	0.007 (0.086)	0.124 (0.352)	54
∆ TG	SCW	0.107^a^	0.011, 0.204	0.006^a^	−0.001, 0.013	0 (0)	0.017 (0.132)	32
∆ TG	SCE	0.060^a^	−0.036, 0.156	0.005^a^	−0.001, 0.01	0 (0)	0.028 (0.167)	49
∆ TG	FLW	0.089^a^	−0.01, 0.2	0.001^a^	−0.008, 0.009	0.007 (0.083)	0.035 (0.187)	58
∆ TG	FLE	0.141^a^	0.062, 0.22	−0.001^a^	−0.004, 0.003	0 (0)	0.022 (0.149)	54
∆ BMI	SCW	−0.009^a^	−0.047, 0.034	−0.002^a^	−0.005, 0.002	0.002 (0.04)	0.002 (0.046)	28
∆ BMI	SCE	0.021^a^	−0.019, 0.062	−0.001^a^	−0.004, 0.002	0.001 (0.026)	0.005 (0.068)	47
∆ BMI	FLW	−0.017^a^	−0.067, 0.037	−0.004^a^	−0.008, 0	0.002 (0.04)	0.008 (0.09)	55
∆ BMI	FLE	−0.015^a^	−0.073, 0.042	−0.003^a^	−0.006, 0	0 (0.009)	0.012 (0.108)	54
**Log (residual yolk)**	SCW	0.438^ab^	0.08, 0.771	0.038^a^	0.009, 0.066	0.025 (0.158)	0.201 (0.448)	31
**Log (residual yolk)**	SCE	0.149^b^	−0.031, 0.316	0.026^a^	0.013, 0.04	0.015 (0.121)	0.087 (0.296)	50
**Log (residual yolk)**	FLW	0.449^ab^	0.307, 0.607	0.007^a^	−0.01, 0.025	0.074 (0.271)	0.065 (0.255)	58
**Log (residual yolk)**	FLE	0.594^a^	0.457, 0.735	0.017^a^	0.006, 0.029	0.028 (0.167)	0.065 (0.255)	54
**Log(fat body)**	SCW	−0.558^ab^	−0.805, −0.328	0.018^a^	−0.005, 0.039	0.018 (0.134)	0.095 (0.308)	31
**Log(fat body)**	SCE	−0.766^b^	−0.877, −0.659	0.017^a^	0.007, 0.027	0.01 (0.101)	0.035 (0.186)	50
**Log(fat body)**	FLW	−0.691^ab^	−0.79, −0.584	0.007^a^	−0.005, 0.019	0.031 (0.175)	0.032 (0.18)	58
**Log(fat body)**	FLE	−0.528^a^	−0.644, −0.415	0.004^a^	−0.006, 0.015	0.027 (0.163)	0.044 (0.209)	54
**Duration**	SCW	−14.157^a^	−15.223, −13.149	0.018^a^	−0.059, 0.094	0.076 (0.276)	1.942 (1.394)	32
**Duration**	SCE	−13.067^a^	−13.51, −12.622	0.027^a^	−0.03, 0.084	0.731 (0.855)	0.571 (0.756)	50
**Duration**	FLW	−10.855^b^	−11.95, −9.713	0.054^a^	−0.037, 0.144	0.801 (0.895)	3.465 (1.862)	53
**Duration**	FLE	−11.076^b^	−12.016, −10.144	0.043^a^	−0.086, 0.169	2.453 (1.566)	2.256 (1.502)	45

Abbreviation: SD, Standard deviation.

We next examined the extent to which morphological phenotypes varied across populations within a temperature, including whether differences in the influence of incubation temperature were driven by variation at 33.5°C, 29.5°C, or both by comparing model means under a common egg mass. There were significant differences in trait values between at least two populations for all morphological traits after controlling for egg mass differences, with interpopulation variation in morphological traits occurring primarily at 29.5°C (Appendix). For instance, the influence of incubation temperature on mass of SCE hatchlings was primarily driven by a reduction in mass at 29.5°C relative to the other populations (SCW: *x̄* = 51.41, CI = [48.60, 54.22]; SCE: *x̄* = 47.49, CI = [46.48, 48.50]; FLW: *x̄* = 51.54, CI = [49.38, 53.69]; FLE: *x̄* = 52.80, CI = [51.16, 54.45]; Figure 2b). This pattern was mostly consistent across additional traits that were differentially impacted by incubation temperature. Both HL (SCW: *x̄* = 35.78, CI = [34.89, 36.67]; SCE: *x̄* = 34.46, CI = [34.12, 34.81]; FLW: *x̄* = 35.87, CI = [35.41, 36.34]; FLE: *x̄* = 36.90, CI = [36.47, 37.32]) and HW (SCW: *x̄* = 21.10, CI = [20.38, 21.83]; SCE: *x̄* = 20.01, CI = [19.72, 20.29]; FLW: *x̄* = 20.82, CI = [20.42, 21.22]; FLE: *x̄* = 21.24, CI = [21.01, 21.47]) were reduced at SCE relative to the other populations and TG was reduced at SCE relative to FLE (SCE: *x̄* = 4.54, CI = [4.44, 4.64]; FLE: *x̄* = 4.93, CI = [4.82, 5.04]). The exception was SVL, which appeared to involve differences at both 29.5°C (SCW: *x̄* = 11.88, CI = [11.45, 12.31]; SCE: *x̄* = 11.57, CI = [11.38, 11.75]; FLW: *x̄* = 11.88, CI = [11.61, 12.16]; FLE: *x̄* = 12.12, CI = [11.93, 12.31]) and 33.5°C (SCW: *x̄* = 12.32, CI = [12.01, 12.63]; SCE: *x̄* = 12.37, CI = [12.21, 12.53]; FLW: *x̄* = 12.02, CI = [11.79, 12.25]; FLE: *x̄* = 12.23, CI = [12.06, 12.40]; Figure 2c). Ratios of morphological traits to hatchling mass showed no significant differences across populations at either temperature.

### 3.3 Metabolic traits

**FIGURE 3:**
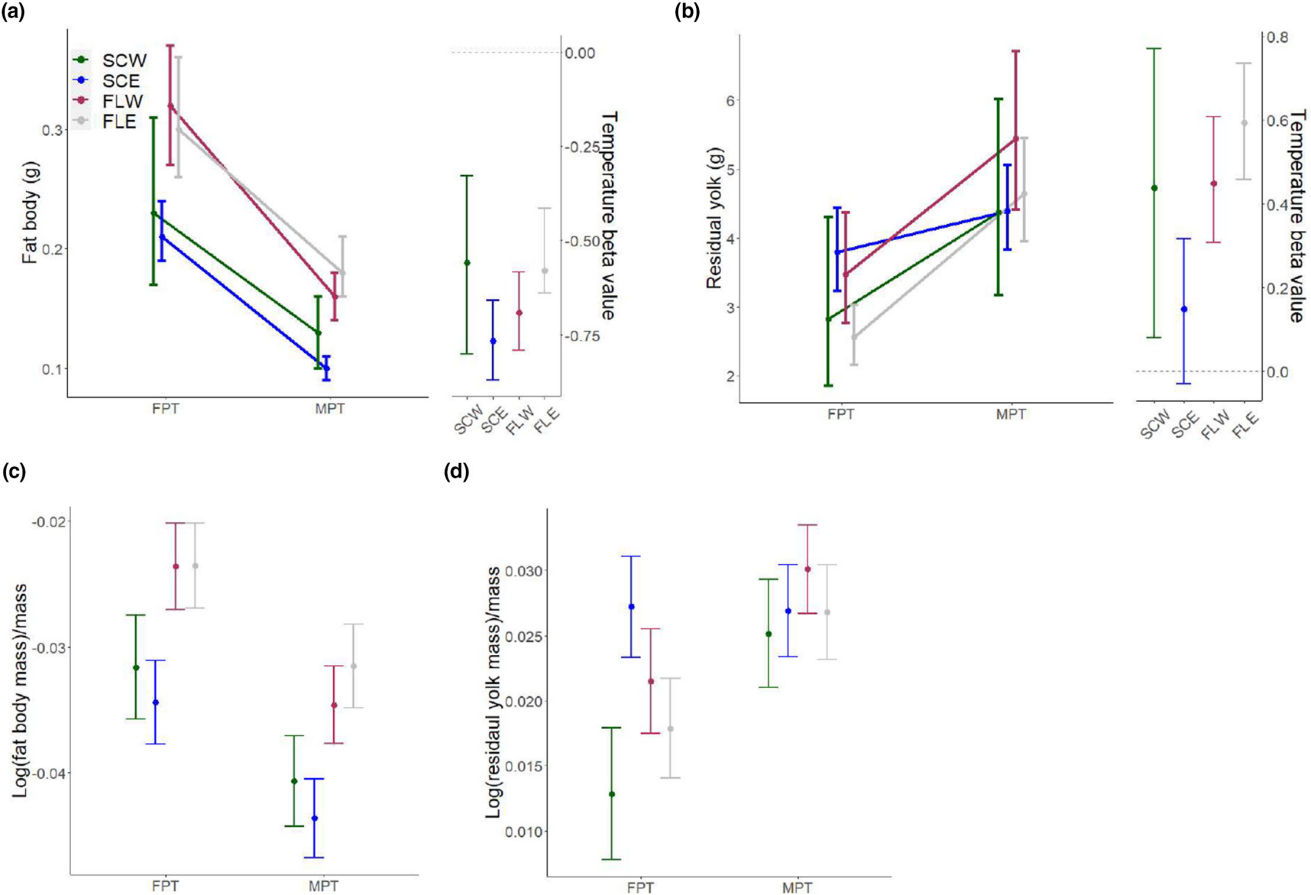
Population variation in metabolic traits and the influence of incubation temperature, showing (a) fat body mass, (b) residual yolk mass, (c) mass‐specific fat body mass, and (d) mass‐specific residual yolk mass. Beta values are effect size estimates, and error bars represent 95% confidence intervals.

As with morphological traits, we also examined the effect of egg mass and incubation temperature on metabolic traits across populations. As egg mass increased, ∆mass decreased at the two southern populations, but had no effect in northern populations (Table 2). However, comparison of beta values across sites showed only a significant difference between FLW and SCW (FLW: *β* = −0.086, CI = [−0.120, −0.053]; SCW: *β* = 0.009, CI = [−0.048, 0.065]). A positive influence of egg mass on residual yolk mass was observed across all populations except for FLW, but differences across populations were not significant. Incubation temperature did not affect ∆mass or ∆BMI at any population, but exerted negative influences on ∆SVL at SCE and fat body mass at all populations (Table 2, Figure 3a). On the other hand, there was a significantly positive influence of incubation temperature on ∆TG and residual yolk mass (Table 2, Figure 3b) in at least one population. Whereas the effect sizes of temperature on ∆SVL and ∆TG did not differ across sites, the influence of temperature on residual yolk mass and fat body mass did. Compared to FLE, the influence of temperature was larger at SCE for fat body mass (SCE: *β* = −0.766, CI = [−0.877, −0.659]; FLE: *β* = −0.528, CI = [−0.644, −0.415]; Table 2; Figure 3a) while the opposite was true for residual yolk mass (SCE: *β* = 0.149, CI = [−0.031, 0.316]; FLE: *β* = 0.594, CI = [0.457, 0.735]; Table 2; Figure 3b).

When comparing metabolic phenotypes across populations after correcting for egg mass, we found significant differences in fat body mass between SCE and both FLE and FLW at 29.5°C (SCW: *x̄* = 0.23, CI = [0.17, 0.31]; SCE: *x̄* = 0.21, CI = [0.19, 0.24]; FLW: *x̄* = 0.32, CI = [0.27, 0.37]; FLE: *x̄* = 0.30, CI = [0.26, 0.36]) and 33.5°C (SCW: *x̄* = 0.13, CI = [0.10, 0.16]; SCE: *x̄* = 0.10, CI = [0.09, 0.11]; FLW: *x̄* = 0.16, CI = [0.14, 0.18]; FLE: *x̄* = 0.18, CI = [0.15, 0.21]), with a trend for smaller fat body masses at the northern populations (Figure 3a; Appendix). Consistent with the decreased influence of incubation temperature on residual yolk mass at SCE, animals from 29.5°C at SCE had significantly higher residual yolk mass compared to FLE (SCE: *x̄* = 3.76, CI = [3.21, 4.40]; FLE: *x̄* = 2.55, CI = [2.15, 3.02]; Figure 3b; Appendix). Upon examination of the mass‐corrected allocation of maternal resources toward metabolic phenotypes, there were significant differences for both residual yolk mass and fat body mass across populations. Animals from the southern populations tended to allocate more resources toward fat body mass than the northern populations at both 29.5°C (SCW: *x̄* = 0.0045, CI = [0.0036, 0.0054]; SCE: *x̄* = 0.0045, CI = [0.0038, 00052]; FLW: *x̄* = 0.0063, CI = [0.0055, 0.0070]; FLE: *x̄* = 0.0061, CI = [0.0054, 0.0068]) and 33.5°C (SCW: *x̄* = 0.0023, CI = [0.0016, 0.0031]; SCE: *x̄* = 0.0018, CI = [0.0012, 00025]; FLW: *x̄* = 0.0030, CI = [0.0024, 0.0036]; FLE: *x̄* = 0.0033, CI = [0.0026, 0.0039]; Figure 3c), and animals from SCE at 29.5°C allocated more resources toward residual yolk mass relative to SCW and FLE (SCW: *x̄* = 0.0129, CI = [0.0078, 0.0179]; SCE: *x̄* = 0.0272, CI = [0.0233, 0.0311]; FLW: *x̄* = 0.0215, CI = [0.0175, 0.0255]; FLE: *x̄* = 0.0179, CI = [0.0140, 0.0217]; Figure 3d).

**FIGURE 4:**
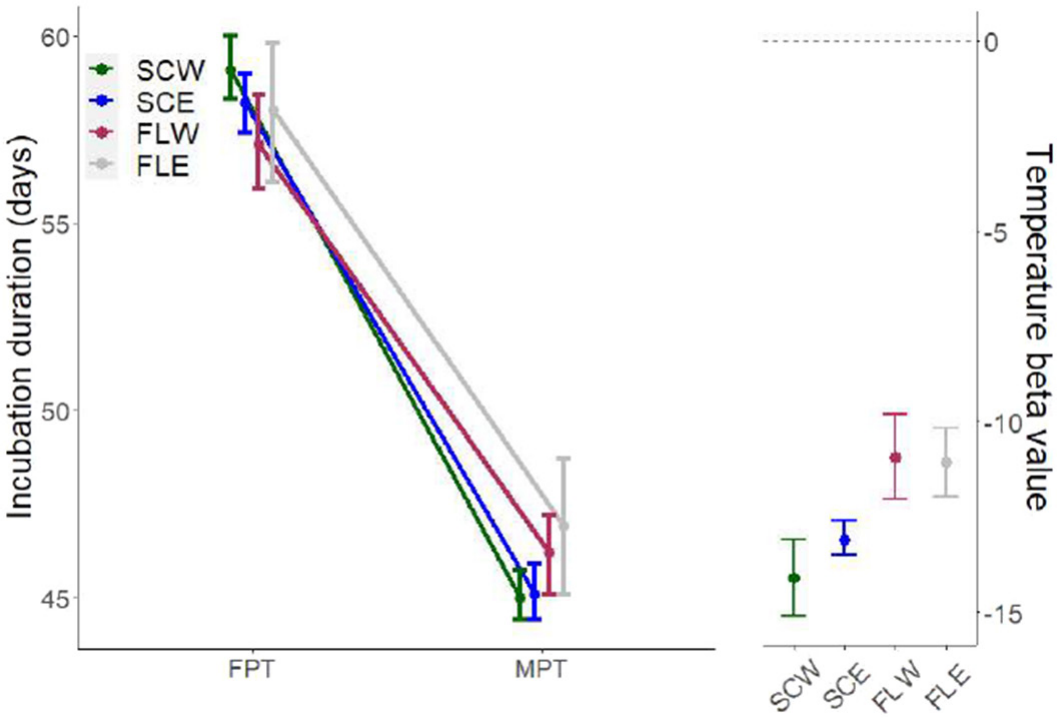
Population variation in the influence of incubation temperature on incubation duration. Beta values are effect size estimates, and error bars represent 95% confidence intervals.

There was no significant influence of egg mass on incubation duration at any population, whereas incubation temperature had a negative influence on incubation duration across all sites (Figure 4). The influence of temperature was greater at the northern populations than at the southern populations (SCW: *β* = −14.16, CI = [−15.22, −13.15]; SCE: *β* = −13.07, CI = [−13.51, −12.62]; FLW: *β* = −10.86, CI = [−11.95, −9.71]; FLE: *β* = −11.08, CI = [−12.02, −10.14]), driven by comparatively shorter incubation periods at 33.5°C (SCW: *x̄* = 45.19, CI = [44.30, 46.08]; SCE: *x̄* = 45.18, CI = [44.52, 45.85]; FLW: *x̄* = 46.05, CI = [45.14, 46.96]; FLE: *x̄* = 46.79, CI = [45.45, 48.14]) and longer incubation periods at 29.5°C (SCW: *x̄* = 59.34, CI = [58.12, 60.57]; SCE: *x̄* = 58.25, CI = [57.56, 58.94]; FLW: *x̄* = 56.91, CI = [55.70, 58.11]; FLE: *x̄* = 57.87, CI = [56.45, 59.29]; Figure 4). However, differences across sites within temperatures were not significant (Appendix).

## 4 DISCUSSION

Patterns of population‐level variation in embryonic responses to maternal provisioning and environmental factors have the potential to inform how the developmental environment contributes to evolutionary change. We observed that, generally, the influence of maternal provisioning on hatchling traits did not vary across populations; however, incubation temperature exerted population‐specific effects on both morphological and metabolic traits. This may be explained by a constrained relationship between egg mass and hatch mass (Deeming & Birchard, 2007), which is expected to be under strong selection as hatchling mass is often an important component of survival and fitness (Ronget et al., 2018; Stearns, 2000). Rather than alter this relationship, selection instead tends to act on aspects of maternal allocation, such as egg size and number, to best match population‐specific conditions (Angilletta et al., 2004; Sinervo, 1990). On the other hand, responses to incubation temperature may be in part the result of differences in natural nest temperatures across populations (Bock et al., 2020; Du et al., 2019). Such differences likely select for embryonic responses to temperature that reduce developmental cost and decrease the need to acquire resource prior to colder, harsher winters at northern latitudes (Pettersen et al., 2023). Our results suggest that plastic responses to incubation temperature, but not maternal provisions, vary across populations and have potential to be modified by selection.

The four populations examined in this study encompassed a large proportion of the alligator's latitudinal range, with two populations from the northern extent and two populations from the southern extent. While not statistically significant, we observed a trend for smaller egg masses at the northern populations relative to the southern populations. In crocodylians, egg mass scales with maternal body size (Larriera et al., 2004), and differences in maternal size might underlie population differences observed here. In mammals, animals from high latitudes tend to be larger than those from low latitudes in a pattern known as Bergmann's rule (Blackburn et al., 1999), and while this seems to hold in turtles and birds, it does not in other reptiles, such as squamates (Ashton, 2002; Ashton & Feldman, 2003) and has not been examined in crocodylians. On the other hand, trade‐offs between offspring size and number have been shown to vary, with fewer, larger offspring favored in colder environments and later in the reproductive season (Angilletta et al., 2004; Hall et al., 2020). Nonetheless, larger egg sizes at southern populations do not support either of these hypotheses. Alternatively, allometric relationships between maternal size and egg mass can be altered by environmental conditions, such as salinity stress (Murray et al., 2013). Given the lack of information on nesting females here, it remains unknown whether differences in egg size are the result of variation in maternal size across populations (maximum size or age at reproduction), population‐specific allometric relationships, or differences in maternal allocation and is an interesting area of future research.

We hypothesized that northern populations would show evidence of adaptation to cooler environments by altering embryonic responses to temperature, resulting in faster development and increased mass and growth at cool incubation temperatures. However, only a few traits showed evidence of latitudinal patterns. We found that incubation duration was more strongly influenced by incubation temperature at the northern populations relative to the southern populations. Specifically, embryos from northern populations developed slightly slower at cooler temperatures and faster at warmer temperatures compared to southern populations. Latitudinal differences in incubation duration have been shown in several species and generally follow one of two patterns: co‐gradient variation, in which cooler population development more slowly relative to warmer populations and counter‐gradient variation, in which cooler populations development more quickly than warmer populations (Conover & Schultz, 1995; Pettersen, 2020). While our differences within temperatures were not significant, they followed patterns of both co‐gradient variation (at 29.5°C) and counter‐gradient variation (at 33.5°C), which only partially support our predictions. Similar results have been shown in Asian pond turtles (*Mauremys mutica*; Zhao et al., 2015) and may suggest that the mechanisms responsible for variation in incubation duration across populations are temperature specific. Alternatively, increased plasticity of developmental rate at northern populations may allow embryos to take advantage of warm conditions when they do arise under natural thermal regimes, reducing development time and the cost of development and resulting in earlier hatching. Additional experiments incorporating more incubation treatments and populations are needed to more completely discern how the relationship between temperature and developmental rate differs across populations as well as the underlying mechanisms responsible. We also observed that southern populations tended to allocate more resources toward fat body mass than northern populations at both incubation temperatures, opposite our predictions. The role of the fat body in alligators is not known, and further work examining its function, including how fat body size/mass early in life might impact survival and later life fitness, is needed to more fully appreciate the potential consequences of this pattern.

Apart from latitudinal trends, there were several differences in the influence of incubation temperature between population pairs, specifically between SCE and other populations and primarily driven by temperature's influence on hatchling mass. In alligators, animals incubated at 33.5°C have been previously shown to be larger in mass than those at 29.5°C (Bock et al., 2021), which was upheld across all of our populations. However, at SCE, the reduction of hatchling mass at 29.5°C was particularly pronounced and appeared to drive additional phenotypic differences. Hatchling mass relative to egg mass reflects the efficiency by which maternal resources are converted into hatchling tissue and is likely a product of the energetic cost of embryonic development (Pettersen et al., 2019). The reduction in mass at SCE at 29.5°C relative to the other sites suggests that development at SCE was particularly inefficient at 29.5°C. Across our populations, SCE is the only coastal site, which may put additional stressors on embryos and breeding females (Albecker & McCoy, 2017). Indeed, the salinity of the incubation environment has been shown previously to have a negative effect on hatchling mass (Bower et al., 2013). However, we only saw an effect at 29.5°C, and while differential responses to incubation temperature under salinity stress have been reported (e.g., Hudak & Dybdahl, 2023), the extent to which egg yolks from SCE have increased salinity, if at all, relative to our other populations is unknown. Interestingly, animals incubated at 29.5°C at SCE also tended to have residual yolk reserves that were larger or equivalent to other populations after controlling for mass. This may be driven by a reduced rate of yolk assimilation during development or may suggest an increased importance of residual yolk mass under cooler temperatures at SCE, despite reduction in overall size (Murphy et al., 2020; Radder et al., 2004).

The lack of latitudinal trends in most of the morphological and metabolic traits examined here suggests that latitude may not be the best or only microclimatic proxy within which to understand variation in responses to the developmental environment, particularly incubation temperature. A similar lack of latitudinal patterns in response to incubation temperature was shown across several populations of painted turtles (*Chrysemys picta*), another TSD species (Bodensteiner et al., 2019). These results may be driven by microhabitat population differences in temperature that are not represented by latitude. On the other hand, maternal nest site choice can be an important driver of nest temperatures and may vary across populations (Du et al., 2023; Warner & Shine, 2008). This can result in similar nest temperatures despite different environmental temperatures (Bodensteiner et al., 2023) and would reduce or eliminate selective pressures for differential responses to incubation temperature. More work is needed to understand how nest temperatures vary across the alligator's range and the role of maternal nest site choice. Another possible reason for the lack a latitudinal patterns is population‐specific, non‐thermal microclimatic variables (i.e., salinity) that can influence thermal reaction norms. Additionally, other maternal effects, such as yolk composition and deposition of hormones and anthropogenic contaminants, may, in addition to temperature, influence phenotype (Bae et al., 2021; Du, Ji, et al., 2010; Groothuis et al., 2005), but were not considered here. Furthermore, since our design focused on incubation temperatures that produce nearly 100% males or females, population variation at each temperature may have been driven by sex differences that would not be explained by latitude. While previous work has shown that phenotypic differences between incubation temperatures are the result of temperature and not sex (Bock et al., 2023), whether sex differences exist across populations irrespective of temperature remains an open question, future work examining the latter and the role of additional aspects of the developmental environment as potential drivers of variable responses to temperature across populations and the consistency of such effects across years will be particularly informative.

One important component not examined in this study is the role of genetics in shaping trait variation across populations. Specifically, high gene flow between populations can limit the ability of selection to drive local adaptation, rendering the differences observed across our populations unlikely to have a genetic basis or be adaptive (Kawecki & Ebert, 2004; Stamp & Hadfield, 2020). Limited information on population structure of alligators exists, but work utilizing microsatellites has shown that populations generally follow an isolation by distance model: genetic differences between FLW and FLE are relatively low, forming a group with other FL and GA populations, but separate from Louisiana and Texas populations (Davis et al., 2002; Ryberg et al., 2002). Meanwhile, one population examined in SC (Santee Coastal Reserve) was shown to be genetically distinct from both of the latter groups (Davis et al., 2002). These results suggest that there is gene flow between FLW and FLE but limited connectively between them and our northern populations. Given this information, it is likely that FLE and FLW are more closely related genetically than to SCW or SCE, and that genetic distances between FLW and FLE are likely reduced relative to those between SCW and SCE. This aligns with our results as we observed differences in both incubation duration and mass‐corrected fat body mass between northern and southern population pairs. Furthermore, while there were no differences between FLE and FLW for any trait, SCE differed from all other populations in response to temperature for mass, showing additional population‐specific differences in other traits, usually between SCE and a southern population. However, further work on the genetic structure of these populations is needed to understand the genetic basis of the differences observed, which is critical if they are to be adaptive or modified by selection.

## 5 CONCLUSIONS

Overall, we found variation in developmental plasticity to incubation temperature for morphological and metabolic phenotypes across populations of alligators. In contrast, the influence of maternal provisioning on hatchling traits was mostly consistent across populations. While the adaptive value of variable plastic responses to incubation temperature was not explicitly tested, variation across populations may suggest evolutionary potential. However, the lack of information on environmental differences between populations, differential selective pressures acting on hatchling alligators, and the genetic basis of the differences observed prevent drawing broad conclusions. Determining the causes of these differences, including the developmental mechanisms involved, would provide important insight into how components of the developmental environment and embryonic responses to them influence intraspecific variation and may contribute to adaptive evolutionary change.

## AUTHOR CONTRIBUTIONS

**Christopher R. Smaga:** Conceptualization (equal); formal analysis (lead); investigation (lead); writing – original draft (lead). **Samantha L. Bock:** Investigation (equal); writing – review and editing (equal). **Josiah M. Johnson:** Investigation (equal); writing – review and editing (equal). **Thomas Rainwater:** Investigation (supporting); writing – review and editing (equal). **Randeep Singh:** Investigation (supporting); writing – review and editing (equal). **Vincent Deem:** Investigation (supporting); writing – review and editing (equal). **Andrew Letter:** Investigation (supporting); writing – review and editing (equal). **Arnold Brunell:** Investigation (supporting); writing – review and editing (equal). **Benjamin Parrott:** Conceptualization (equal); funding acquisition (lead); investigation (equal); resources (lead); supervision (lead); writing – review and editing (equal).

## CONFLICT OF INTEREST STATEMENT

Authors declare no competing interests.

### OPEN RESEARCH BADGES

This article has earned an Open Data badge for making publicly available the digitally‐shareable data necessary to reproduce the reported results. The data is available at https://doi.org/10.5061/dryad.280gb5mvk.

## DISCLAIMER

This report was prepared as an account of work sponsored by an agency of the US Government. Neither the US Government nor any agency thereof, nor any of their employees, make any warranty, express or implied, or assume any legal liability or responsibility for the accuracy, completeness, or usefulness of any information, apparatus, product, or process disclosed, or represent that its use would not infringe privately owned rights. Reference herein to any specific commercial product, process, or service by trade name, trademark, manufacturer, or otherwise does not necessarily constitute or imply its endorsement recommendation, or favoring by the US Government or any agency thereof. The views and opinions of authors expressed herein do not necessarily state or reflect those of the US Government or any agency thereof.

## Data Availability

Data for the manuscript are available on dryad (https://datadryad.org/stash/share/Ee6Qwq0ccV3fJku7nMFdkJySnmvDiDoMGuyug‐J75Lo).

## Predicted mean values of traits at a common egg mass (82.75 g).

**Table:**

Site	Phenotype	Temp (°C)	Mean	CI
SCW	Mass	29.5	51.41	48.60, 54.22
SCW	Mass	33.5	53.63	51.58, 55.68
SCE	Mass	29.5	47.49	46.48, 48.50
SCE	Mass	33.5	54.44	53.56, 55.31
FLW	Mass	29.5	51.54	49.38, 53.69
FLW	Mass	33.5	54.29	52.39, 56.19
FLE	Mass	29.5	52.80	51.16, 54.45
FLE	Mass	33.5	56.04	54.53, 57.56
SCW	SVL	29.5	11.88	11.45, 12.31
SCW	SVL	33.5	12.32	12.01, 12.63
SCE	SVL	29.5	11.57	11.38, 11.75
SCE	SVL	33.5	12.37	12.21, 12.53
FLW	SVL	29.5	11.88	11.61, 12.16
FLW	SVL	33.5	12.02	11.79, 12.25
FLE	SVL	29.5	12.12	11.93, 12.31
FLE	SVL	33.5	12.23	12.06, 12.40
SCW	TG	29.5	4.74	4.52, 4.96
SCW	TG	33.5	4.70	4.54, 4.86
SCE	TG	29.5	4.54	4.44, 4.64
SCE	TG	33.5	4.73	4.64, 4.81
FLW	TG	29.5	4.80	4.63, 4.96
FLW	TG	33.5	4.77	4.63, 4.92
FLE	TG	29.5	4.93	4.82, 5.04
FLE	TG	33.5	4.84	4.74, 4.94
SCW	Head length	29.5	35.78	34.89, 36.67
SCW	Head length	33.5	36.50	35.86, 37.14
SCE	Head length	29.5	34.46	34.12, 34.81
SCE	Head length	33.5	35.84	35.55, 36.13
FLW	Head length	29.5	35.87	35.41, 36.34
FLW	Head length	33.5	36.43	36.06, 36.81
FLE	Head length	29.5	36.90	36.47, 37.32
FLE	Head length	33.5	36.90	36.51, 37.29
SCW	Head width	29.5	21.10	20.38, 21.83
SCW	Head width	33.5	20.64	20.12, 21.17
SCE	Head width	29.5	20.01	19.72, 20.29
SCE	Head width	33.5	20.18	19.94, 20.42
FLW	Head width	29.5	20.82	20.42, 21.22
FLW	Head width	33.5	20.60	20.26, 20.94
FLE	Head width	29.5	21.24	21.01, 21.47
FLE	Head width	33.5	20.57	20.37, 20.78
SCW	BMI	29.5	2.14	2.05, 2.22
SCW	BMI	33.5	2.18	2.12, 2.24
SCE	BMI	29.5	2.04	2.00, 2.09
SCE	BMI	33.5	2.21	2.17, 2.25
FLW	BMI	29.5	2.18	2.05, 2.30
FLW	BMI	33.5	2.26	2.15, 2.38
FLE	BMI	29.5	2.17	2.11, 2.24
FLE	BMI	33.5	2.29	2.23, 2.35
SCW	∆ mass	29.5	−2.15	−3.07, −1.2
SCW	∆ mass	33.5	−3.21	−3.86, −2.56
SCE	∆ mass	29.5	−2.63	−3.20, −2.06
SCE	∆ mass	33.5	−3.27	−3.79, −2.75
FLW	∆ mass	29.5	−2.77	−3.21, −2.33
FLW	∆ mass	33.5	−2.88	−3.23, −2.53
FLE	∆ mass	29.5	−2.61	−3.22, −1.99
FLE	∆ mass	33.5	−3.39	−3.97, −2.81
SCW	∆ SVL	29.5	0.71	0.39, 1.03
SCW	∆ SVL	33.5	0.49	0.24, 0.73
SCE	∆ SVL	29.5	0.76	0.58, 0.94
SCE	∆ SVL	33.5	0.48	0.32, 0.65
FLW	∆ SVL	29.5	0.65	0.43, 0.86
FLW	∆ SVL	33.5	0.71	0.54, 0.89
FLE	∆ SVL	29.5	0.64	0.48, 0.80
FLE	∆ SVL	33.5	0.61	0.46, 0.75
SCW	∆ TG	29.5	−0.11	−0.22, 0.01
SCW	∆ TG	33.5	0.00	−0.08, 0.08
SCE	∆ TG	29.5	−0.04	−0.11, 0.04
SCE	∆ TG	33.5	0.02	−0.04, 0.09
FLW	∆ TG	29.5	−0.04	−0.15, 0.06
FLW	∆ TG	33.5	0.05	−0.04, 0.14
FLE	∆ TG	29.5	−0.07	−0.13, −0.01
FLE	∆ TG	33.5	0.07	0.02, 0.13
SCW	∆ BMI	29.5	−0.21	−0.27, −0.15
SCW	∆ BMI	33.5	−0.22	−0.27, −0.17
SCE	∆ BMI	29.5	−0.24	−0.28, −0.20
SCE	∆ BMI	33.5	−0.22	−0.25, −0.18
FLW	∆ BMI	29.5	−0.22	−0.28, −0.17
FLW	∆ BMI	33.5	−0.24	−0.28, −0.20
FLE	∆ BMI	29.5	−0.21	−0.26, −0.17
FLE	∆ BMI	33.5	−0.23	−0.27, −0.19
SCW	Duration	29.5	59.34	58.12, 60.57
SCW	Duration	33.5	45.19	44.30, 46.08
SCE	Duration	29.5	58.25	57.56, 58.94
SCE	Duration	33.5	45.18	44.52, 45.85
FLW	Duration	29.5	56.91	55.70, 58.11
FLW	Duration	33.5	46.05	45.14, 46.96
FLE	Duration	29.5	57.87	56.45, 59.29
FLE	Duration	33.5	46.79	45.45, 48.14
SCW	Residual yolk	29.5	2.79	1.84, 4.24
SCW	Residual yolk	33.5	4.33	3.16, 5.92
SCE	Residual yolk	29.5	3.76	3.21, 4.40
SCE	Residual yolk	33.5	4.36	3.79, 5.02
FLW	Residual yolk	29.5	3.47	2.76, 4.36
FLW	Residual yolk	33.5	5.43	4.40, 6.70
FLE	Residual yolk	29.5	2.55	2.15, 3.02
FLE	Residual yolk	33.5	4.62	3.93, 5.42
SCW	Fat body	29.5	0.23	0.17, 0.31
SCW	Fat body	33.5	0.13	0.10, 0.16
SCE	Fat body	29.5	0.21	0.19, 0.24
SCE	Fat body	33.5	0.10	0.09, 0.11
FLW	Fat body	29.5	0.32	0.27, 0.37
FLW	Fat body	33.5	0.16	0.14, 0.18
FLE	Fat body	29.5	0.30	0.26, 0.36
FLE	Fat body	33.5	0.18	0.15, 0.21
